# Association between triglyceride glucose index and suicide attempts in patients with first-episode drug-naïve major depressive disorder

**DOI:** 10.3389/fpsyt.2023.1231524

**Published:** 2023-07-28

**Authors:** Junjun Liu, Xiaomin Zhu, Yang Liu, Fengnan Jia, Hsinsung Yuan, Qingyuan Wang, Xiaobin Zhang, Zhe Li, Xiangdong Du, Xiangyang Zhang

**Affiliations:** ^1^Nanjing Meishan Hospital, Nanjing, China; ^2^Suzhou Guangji Hospital, The Affiliated Guangji Hospital of Soochow University, Suzhou, China; ^3^Medical College of Soochow University, Suzhou, China; ^4^Clinical Medical Department, The Second Clinical Medical College, Nanjing Medical University, Nanjing, China; ^5^CAS Key Laboratory of Mental Health, Institute of Psychology, Chinese Academy of Sciences, Beijing, China

**Keywords:** triglyceride glucose index, suicide attempts, insulin resistance, major depressive disorder, association

## Abstract

**Objective:**

Triglyceride glucose (TyG) index has been suggested as an alternative indicator of insulin resistance (IR); however, the association between TyG index and suicide attempts (SA) in major depressive disorder (MDD) is unclear. The aim of this study was to investigate the relationship between TyG index and SA in Chinese patients with first-episode drug-naïve (FEDN) MDD.

**Methods:**

This cross-sectional study enrolled 1,718 patients with FEDN MDD aged 34.9 ± 12.4 years from the First Hospital of Shanxi Medical University (Taiyuan, Shanxi Province, China) from September 2016 to December 2018. Multivariable binary logistic regression analysis was used to estimate the association between TyG index and the risk of SA. A two-piecewise linear regression model was used to investigate the threshold effects if non-linearity associations existed. Interaction and stratified analyses were performed based on sex, education, marital status, comorbid anxiety, and psychotic symptoms.

**Results:**

Multivariable logistic regression analysis revealed that TyG index was positively associated with the risk of SA after adjusting for confounders (OR = 1.35, 95% CI: 1.04–1.75, *p* = 0.03). Smoothing plots also showed a nonlinear relationship between TyG index and SA, with the inflection point of TyG index being 9.29. On the right of the inflection point, a positive association between TyG index and SA was detected (OR = 3.47, 95% CI: 1.81 to 6.66, *p* < 0.001), while no significant association was observed on the left side of the inflection point (OR = 1.14, 95% CI: 0.79 to 1.66, *p* = 0.476).

**Conclusion:**

The relationship between TyG index and SA risk was non-linear and exhibited a threshold effect in Chinese patients with FEDN MDD. When TyG index was greater than 9.29, they showed a significant positive correlation.

## 1. Introduction

Major depressive disorder (MDD) is a widespread mental health disease clinically defined by significant and persistent low mood, declining role functioning, a poor quality of life, a high risk of medical comorbidity and mortality, and even a propensity for suicide (1). The 2015 Global Disease Burden Study included 310 illnesses and injuries for study, and depression was identified as the third most common disability cause globally (2). Around 17.3 million people in the United States had at least one major depressive episode in 2017, while 260 million people globally suffered from depression (3). Depression’s disability-adjusted life years (DALYs) have increased by 36.5% in China during the past ten years, moving it up from 15th place in 1990 to 10th place in 2017 (4). Additionally, several investigations have revealed that depression is closely related to cardiovascular diseases, metabolic diseases, diabetes, all-cause mortality, and suicide behaviors (5–8).

The most severe outcome of untreated MDD is suicide. Even after receiving treatment for depression, a previous study found that 15% of patients with MDD died by suicide (9). Suicide rates have been increasing both in China and worldwide, presenting a growing public health issue. In 2019, over 700,000 individuals worldwide died by suicide, which accounted for nearly 1% of all deaths (10). The World Health Organization (WHO)’s Mental Health Action Plan 2013–2030 and the United Nations Sustainable Development Goals 2030 have set an aim to reduce suicide rates by one-third by 2030 (11). It is disturbingly common for people with MDD to attempt suicide, with estimates ranging from 16% to 33.7% having done so at least once in their lifetime (12–16). In addition, suicide attempts (SA) are also known to be the strongest predictor of eventual death by suicide (17). Despite this, the reasons behind SA in patients with MDD are still unclear, highlighting a need for further investigation into associated risk factors.

Reduced insulin responsiveness is referred to as insulin resistance (IR), which is a typical hallmark of type 2 diabetes, hypertension, lipid metabolic problems, and even cardiovascular disease (18). A number of observational studies on the general population have shown that IR-related diabetes is associated with depression (19–21). Epidemiological studies and meta-analyses repeatedly show that people with mental problems are more likely to develop type 2 diabetes mellitus (T2DM) and vice versa, reiterating the reciprocal relationship (22). T2DM has been linked to increased treatment resistance, chronicity, and the occurrence of more severe depressive symptoms, whereas depression exacerbates the negative health outcomes, micro- and macrovascular problems, and death rates among patients with T2DM (22). Interesting research by the European Group for the Study of Resistant Depression (GSRD) links a high body mass index (BMI) to higher rates of suicidality, protracted psychiatric hospitalizations over the course of a person’s lifetime, an earlier onset of major depressive disorder (MDD), and the presence of comorbidities (23). Clinical and genomic studies have revealed a polygenic risk that unites disorders connected to IR and MDD, suggesting overlapping etiopathological processes (24). As likely common pathways, immune-inflammation dysregulation, hypothalamic-pituitary-adrenal axis dysfunction, changes in gut microbiome, and disruptions in brain insulin signaling are identified (25). Notably, insulin can cross the blood-brain barrier and is produced centrally by the choroid plexus. Insulin receptors are found on neurons and glia in the nucleus accumbens, amygdala, and hippocampus, which are involved in mood regulation and cognition (26). Within these areas, neuroprotection, neurogenesis, neurotransmission, and synaptic plasticity are all significantly influenced by the complex network of insulin signaling. Additionally, several antidepressants and diabetes medicines have the potential to improve insulin sensitivity indicators and lessen depressed symptoms (27).

In recent years, a brand-new marker of IR has been discovered: the triglyceride-glucose (TyG) index, which is created by adding fasting plasma glucose and triglycerides. According to research, TyG index is connected to depression, cardiovascular disease, and illness prognosis (21, 28). Shi et al. discovered, for instance, that persons with higher TyG index scores are more prone to have depressed symptoms (29). IR may raise the risk of SA as well as depression. To our knowledge, however, no research has looked at the relationship between TyG index and SA in patients with MDD, particularly in the Chinese community. Therefore, the aim of this study was to evaluate the association between TyG index and SA in a sizable Chinese sample of patients who were first-episode and drug-naive (FEDN) MDD.

## 2. Materials and methods

### 2.1. Study design and participants

**Figure 1:**
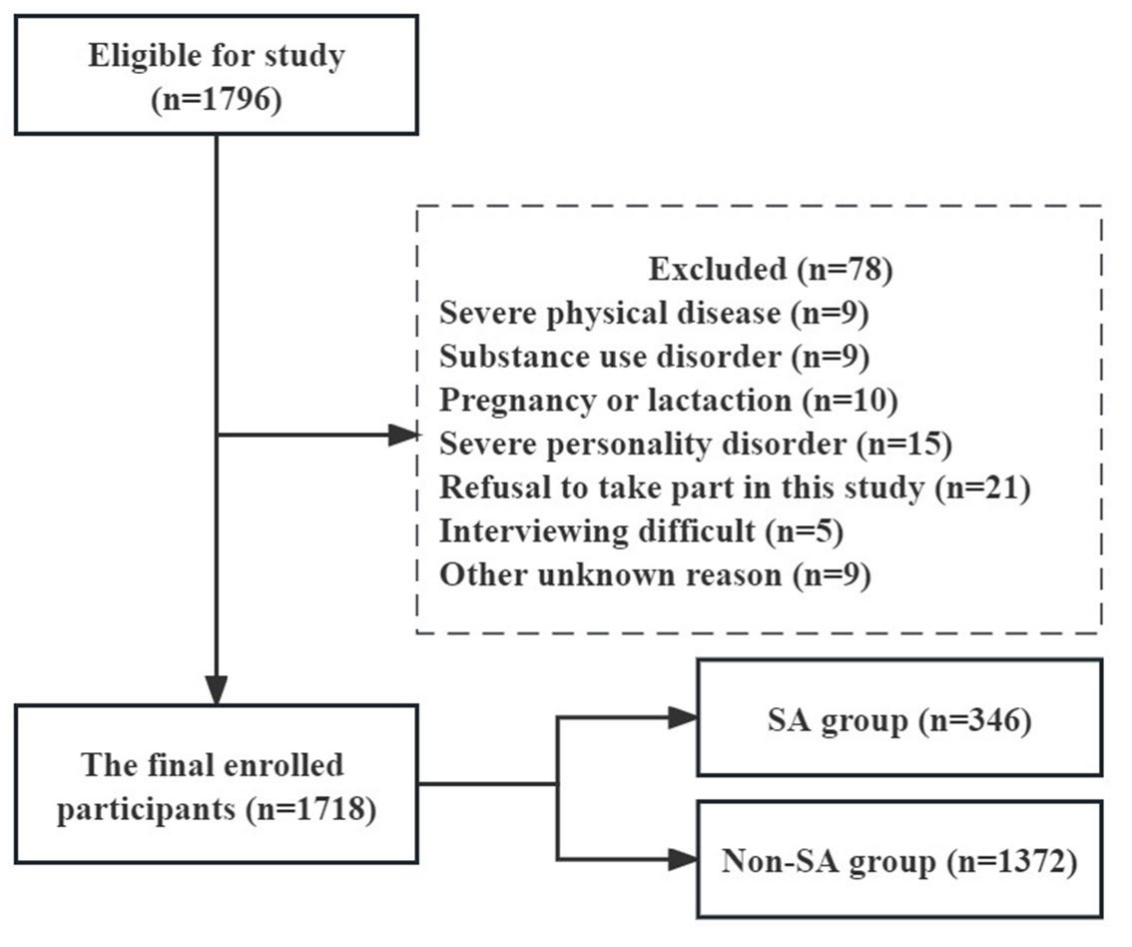
Flow chart of this study.

This cross-sectional study was carried out at the First Hospital of Shanxi Medical University, a general hospital in Taiyuan, Shanxi Province, China, between 2016 and 2018. The patients’ general information and sociodemographic characteristics were gathered using a structured, self-designed questionnaire. Measurements and tests in the medical, psychological, and laboratory domains of the examination were conducted under the supervision of medical professionals. To enhance compliance, each participant also was paid and got a report on their medical results. A total of 1,718 individuals with FEDN MDD (588 males and 1,130 females), who satisfied the Diagnostic and Statistical Manual of Mental Disorders (DSM) IV-TR criteria, were enrolled in the study. The recruitment criteria included: (1) being Han Chinese; (2) being between the ages of 18 and 60; (3) having current depressive symptoms as the first episode; (4) never having received any medication in the past; and (5) having a 17-item HAMD score of ≥24. Exclusion criteria involved having a serious physical illness such as cancer, persistent infection, epilepsy, brain injury, and stroke, being pregnant or breastfeeding women, having alcohol or drug abuse, having severe personality disorder, refusal to take part in the study, unable to engage in interviews owing to a serious clinical condition, and other unspecified factors (shown in Figure 1). All participants completed a written informed consent form before to enrolment, and the study (No. 2016-Y27) was authorized by Shanxi Medical University’s institutional review board (IRB).

### 2.2. Sociodemographic characteristics and anthropometric data

During the visit, the study team collected sociodemographic characteristics and general data from the participants using a standardized questionnaire. Age, gender, education level, marital status, age at sickness beginning, and length of illness were among the details covered by the questionnaire. The categories for educational attainment were junior high school, senior high school, college, and postgraduate. The classification of marital status was either single or married. The systolic (SBP) and diastolic blood pressures (DBP) were calculated as the mean of two readings obtained with a mercury sphygmomanometer on the right arm after lying still for at least 10 min. Weight in kilograms was divided by height in meters squared (kg/m^2^) to determine body mass index (BMI). The following equation was used to generate the TyG index: TyG = Ln [fasting blood glucose (mg/dL) × triglycerides (mg/dL)/2] (30).

Additionally, a face-to-face interview utilizing the question “Have you ever attempted suicide in your lifetime?” was undertaken to get more information about prior SA according to the multiple-center WHO/EURO research (31). Those who responded “yes” were classified as having a history of SA. Then we inquired about the frequency, methodology, and precise dates of suicide attempts from the participants. In cases where their responses were ambiguous or perplexing, supplementary information was sought through interviews with their family members, relatives, or friends. A total of 346 patients with FEDN MDD were documented to have engaged in SA during their initial depressive episode, with 111 of them reporting such attempts within the last month. Among the SA, one patient made four attempts, two patients made three attempts, 26 patients made two attempts, and 317 patients made one attempt. Based on the presence or absence of SA during their initial depressive episode, all subjects were classified into two distinct groups: the group with suicide attempts (SA = 346) and the group without suicide attempts (NSA = 1,372).

### 2.3. Blood samples

Tests were performed on fasting venous blood samples obtained from each patient at the hospital’s laboratory center. Blood biomarkers such as free triiodothyronine (FT3), free thyroxine (FT4), thyroid-stimulating hormone (TSH), thyroid peroxidase antibody (TPOAb), anti-thyroglobulin (TgAb), high-density lipoprotein cholesterol (HDL-C), and low-density lipoprotein cholesterol (LDL-C) were all measured on the same day.

### 2.4. Clinical interview assessments

The 17-item Hamilton Rating Scale (HAMD-17) was used in the study to assess the severity of depression, with a higher score indicating more severe depressed symptoms (32). The Hamilton Anxiety Rating Scale (HAMA) was used to evaluate anxiety symptoms, and a HAMA score of less than 18 indicated comorbid anxiety (33). The Positive and Negative Syndrome Scale (PANSS) was used to measure psychotic symptoms. Patients were deemed to have psychotic symptoms if their total score on the positive subscale was more than or equal to 15 (34). Two certified psychiatrists, each having at least 5 years of clinical experience, were trained to use these rating scales. Repeated evaluations utilizing these scales maintained the inter-observer reliability with a correlation value better than 0.8.

### 2.5. Statistical analysis

Continous variables were presented in various ways depending on how they were distributed. While non-normally distributed data were provided as a median with an interquartile range (IQR), normally distributed variables were expressed as mean with standard deviation (SD). Presented as frequencies and percentages were categorical variables. A one-way ANOVA test, Kruskal–Wallis *H* test, or *χ*^2^ was employed to examine differences between various TyG index quartile groups. The linear relationship between the TyG index and SA was estimated using logistic regression models, and the TyG index was examined as both continuous and categorical variables based on quartiles. 95% confidence intervals (CIs) for the unadjusted and adjusted odds ratios (ORs) were presented. An examination of sensitivity was carried out to guarantee the reliability of the data analysis. In order to determine a *p*-value for trend, the TyG index was transformed into a categorical variable. To assess multicollinearity between independent variables, the variance inflation factor (VIF) was used. Covariates with VIF values higher than 5.0 were not included in the final model. Potential confounders were chosen if they changed the estimates of TyG index on SA by more than 10% or had a *p*-value of less than 0.10 in univariable analysis. Three different models were constructed to verify the stability of the results: an unadjusted model, Model I adjusted for sex and age, and Model II adjusted for sex, age, education, duration of illness, HAMD, HAMA, A-TG, A-TPO, TSH, TC, HDL-c, LDL-c, SBP, and DBP. The non-linear relationship between TyG index and SA was assessed using smoothing plots, and the threshold impact suggested by the smoothing plot was investigated using a two-piecewise linear regression model based on the generalized estimating equation (GEE). Stratified analyses were performed in terms of sex, education, marital status, comorbid anxiety, and psychotic symptoms. The interaction effects in various subgroup variables were evaluated using the log-likelihood ratio test.

All of the analyses were performed using the statistical software packages R (http://www.r-project.org, The R Foundation) and EmpowerStats (http://www.empowerstats.com, X&Y Solution, Inc., Boston, Massachusetts, United States). Statistical significance was defined as two tails of *p* < 0.05. The visuals were created using GraphPad Prism 8.0.

## 3. Results

### 3.1. Baseline characteristics

Figure 1 illustrates the study design, sampling process, and exclusions. This research comprised 1,718 patients with MDD in total. The participants had a mean age of 34.9 ± 12.4 years, and 588 (34.2%) were male. Out of the total participants, 346 (20.1%) patients had SA. Table 1 lists the participant characteristics according to TyG index quartiles. Significant correlations between TyG index quartiles and a number of variables, including sex, HAMD, HAMA, TGAb, TSH, FBG, TC, TG, HDL-c, LDL-c, BMI, SBP, DBP, and SA (all *p* < 0.05), were found.

**Table 1:** Baseline characteristics of participants.

Variables	Total	TyG index quartile				*p*-value
		Q1 (6.6–8.7)	Q2 (8.7–9.1)	Q3 (9.1–9.4)	Q4 (9.4–10.4)	
*N*	1,718	430	429	429	430	
Age (years)	34.9 ± 12.4	34.4 ± 12.4	35.1 ± 12.7	34.8 ± 12.2	35.2 ± 12.5	0.75
Age at onset (years)	34.7 ± 12.3	34.1 ± 12.3	34.9 ± 12.5	34.6 ± 12.1	35.0 ± 12.4	0.73
Duration of illness (months)	5.0 (3.0–8.0)	4.5 (3.0–8.0)	5.0 (3.0–8.0)	5.0 (3.0–8.0)	5.0 (3.0–8.0)	0.12
HAMD	30.3 ± 2.9	29.3 ± 2.9	30.3 ± 2.7	30.4 ± 3.0	31.1 ± 2.9	<0.001
HAMA	20.8 ± 3.5	20.4 ± 3.3	20.4 ± 3.4	20.9 ± 3.4	21.5 ± 3.8	<0.001
TSH (uIU/mL)	5.1 ± 2.6	4.2 ± 2.4	5.0 ± 2.3	5.1 ± 2.4	6.1 ± 2.7	<0.001
TGAb (IU/L)	21.5 (14.4–43.6)	21.2 (14.4–38.5)	19.3 (14.0–42.1)	22.2 (14.4–42.6)	22.7 (15.7–59.1)	0.03
TPOAb (IU/L)	17.4 (12.3–34.6)	18.8 (12.7–31.4)	16.7 (12.1–33.7)	16.4 (12.2–32.3)	20.1 (12.4–41.2)	0.05
FT3 (pmol/L)	4.9 ± 0.7	4.8 ± 0.7	4.9 ± 0.7	4.9 ± 0.7	4.9 ± 0.7	0.17
FT4 (pmol/L)	16.7 ± 3.1	16.9 ± 3.3	16.6 ± 3.0	16.5 ± 3.0	16.8 ± 3.1	0.15
FBG (mmol/L)	5.4 ± 0.7	5.1 ± 0.5	5.4 ± 0.6	5.4 ± 0.6	5.7 ± 0.7	<0.001
TC (mmol/L)	5.3 ± 1.1	4.8 ± 1.0	5.2 ± 1.0	5.3 ± 1.1	5.7 ± 1.1	<0.001
HDL-c (mmol/L)	1.2 ± 0.3	1.3 ± 0.3	1.2 ± 0.3	1.2 ± 0.3	1.1 ± 0.3	<0.001
TG (mmol/L)	2.2 ± 1.0	1.2 ± 0.3	1.7 ± 0.3	2.4 ± 0.3	3.4 ± 0.9	<0.001
LDL-c (mmol/L)	3.0 ± 0.9	2.8 ± 0.8	3.0 ± 0.8	3.0 ± 0.9	3.0 ± 0.9	<0.001
BMI (kg/m^2^)	24.4 ± 1.9	24.1 ± 1.8	24.3 ± 2.1	24.4 ± 1.9	24.6 ± 1.9	0.01
Systolic blood pressure (mmHg)	119.5 ± 10.9	117.7 ± 10.9	118.8 ± 10.7	120.0 ± 10.8	121.4 ± 10.9	<0.001
Diastolic blood pressure (mmHg)	76.0 ± 6.7	75.3 ± 6.7	75.6 ± 6.4	76.1 ± 6.8	76.8 ± 6.9	0.01
Gender						0.04
Male	588 (34.2%)	143 (33.3%)	171 (39.9%)	137 (31.9%)	137 (31.9%)	
Female	1,130 (65.8%)	287 (66.7%)	258 (60.1%)	292 (68.1%)	293 (68.1%)	
Education						0.52
Junior high school	413 (24.0%)	98 (22.8%)	96 (22.4%)	102 (23.8%)	117 (27.2%)	
Senior high school	760 (44.2%)	182 (42.3%)	204 (47.6%)	188 (43.8%)	186 (43.3%)	
College	449 (26.1%)	123 (28.6%)	111 (25.9%)	113 (26.3%)	102 (23.7%)	
Postgraduate	96 (5.6%)	27 (6.3%)	18 (4.2%)	26 (6.1%)	25 (5.8%)	
Marital status						0.97
Single	502 (29.2%)	127 (29.5%)	125 (29.1%)	128 (29.8%)	122 (28.4%)	
Married	1,216 (70.8%)	303 (70.5%)	304 (70.9%)	301 (70.2%)	308 (71.6%)	
Suicide attempts						<0.001
No	1,372 (79.9%)	362 (84.2%)	346 (80.7%)	349 (81.4%)	315 (73.3%)	
Yes	346 (20.1%)	68 (15.8%)	83 (19.3%)	80 (18.6%)	115 (26.7%)	

The variables are presented as *n* (%) or the mean ± SD or median (quartile 1-quartile 3), TyG, triglyceride glucose; HAMD, 17-item Hamilton Rating Scale for Depression; HAMA, 14-item Hamilton Anxiety Rating Scale; TSH, thyroid-stimulating hormone; TGAb, thyroglobulin antibody; TPOAb, thyroid peroxidase antibody; FT3, free triiodothyronine; FT4, free thyroxine; FBG, fasting blood glucose; TC, total cholesterol; HDL-c, high-density lipoprotein cholesterol; TG, triglyceride; LDL-c, low-density lipoprotein cholesterol; BMI, body mass index.

### 3.2. Associations between SA and TyG index

A greater TyG index was substantially linked to a higher risk of SA, according to the fully adjusted data (Table 2; OR = 1.35, 95% CI: 1.04 to 1.75; *p* = 0.03). Patients in the fourth quartile of TyG index showed a greater likelihood of developing SA after controlling for a number of variables (OR = 1.57, 95% CI: 1.11 to 2.23, *p* = 0.01) compared to those in the first quartile.

**Table 2:** Relationship between TyG index and suicide attempts in different models.

Variable	Unadjusted model		Model I		Model II	
	OR (95%CI)	*p*-value	OR (95%CI)	*p*-value	OR (95%CI)	*p*-value
TyG index	1.64 (1.27, 2.11)	<0.001	1.63 (1.26, 2.09)	0.01	1.35 (1.04, 1.75)	0.03
TyG index quartile						
Q1 (6.6–8.7)	Reference		Reference		Reference	
Q2 (8.7–9.1)	1.28 (0.90, 1.82)	0.17	1.28 (0.90, 1.82)	0.18	1.23 (0.86, 1.77)	0.26
Q3 (9.1–9.4)	1.22 (0.86, 1.74)	0.27	1.21 (0.85, 1.73)	0.28	1.05 (0.73, 1.52)	0.79
Q4 (9.4–10.4)	1.94 (1.39, 2.72)	<0.001	1.93 (1.38, 2.70)	<0.001	1.57 (1.11, 2.23)	0.01
*p* for trend	0.004		0.004		0.005	

CI, confidence interval; unadjusted model adjusted for none; Model I adjusted for age, sex; Model II adjusted for age, sex, education, duration of illness, HAMD, HAMA, TSH, A-TG, A-TPO, TC, HDL-c, LDL-c, SBP, DBP.

**Figure 2:**
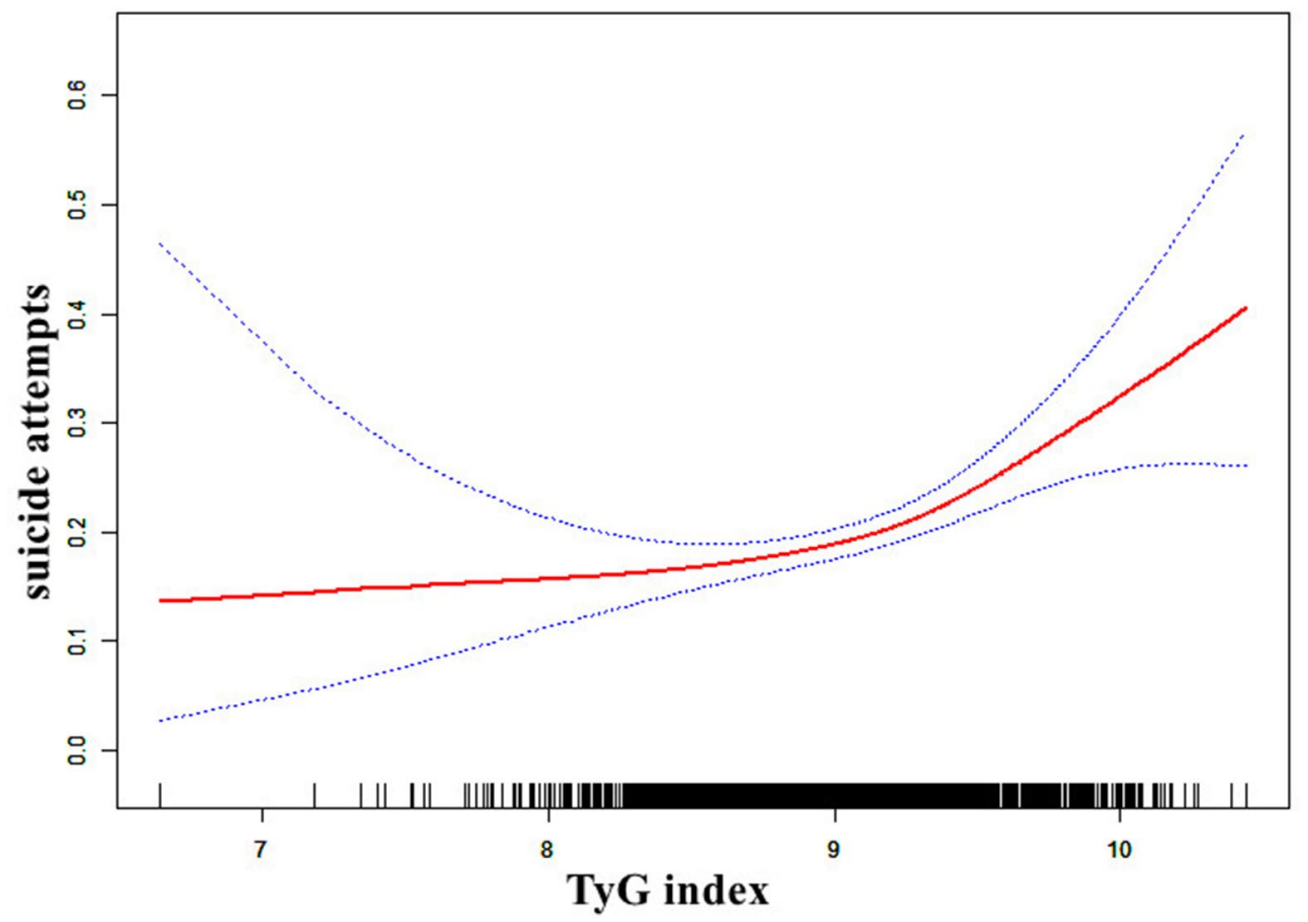
The relationship between TyG index and the probability of suicide attempts. A nonlinear relationship between TyG index and the probability of SA was observed after adjusting for age, sex, education, duration of illness, HAMD, HAMA, TSH, A-TG, A-TPO, TC, HDL-c, LDL-c, SBP, DBP (*p* for non-linearity <0.05).

Figure 2 illustrates the non-linear relationship between TyG index and SA using generalized additive models (*p* for non-linearity <0.05). A two-segment logistic regression model identified an inflection point value of 9.29 for TyG index. Using generalized additive models, Figure 2 shows the non-linear connection between TyG index and SA (*p* for non-linearity <0.05). A two-segment logistic regression model identified an inflection point value of 9.29 for TyG index. For each unit rise in TyG index on the right side of the inflection point, the probability of SA rose substantially by 247% (OR = 3.47, 95% CI: 1.81 to 6.66, *p* < 0.001). On the left side of the inflection point, however, there was no evidence of a significant relationship between TyG index and SA (OR = 1.14, 95% CI: 0.97 to 1.66, *p* = 0.48), as shown in Table 3. 95% confidence intervals (CIs) were calculated using bootstrap techniques around the TyG index’s inflection point, which was 9.15 to 9.39. There were 548 patients with a TyG score more than or equal to 9.29, and 1,170 patients with a TyG index less than 9.29.

**Table 3:** The results of two-piecewise logistic regression model.

Inflection point of TyG index	OR	95% CI	*p*-value
Inflection point	9.29	9.15 to 9.39	
<9.29 slope 1 (*n* = 1,170)	1.14	0.79 to 1.66	0.48
≥9.29 slope 2 (*n* = 548)	3.47	1.81 to 6.66	<0.001
Slope 2 − slope 1	3.03	1.25 to 7.36	0.01
Predicted at 9.29	−1.46	−1.66 to −1.25	
Log likelihood ratio test			0.02

Effect: suicide attempts; cause: TyG index; adjusted for age, sex, education, duration of illness, HAMD, HAMA, TSH, A-TG, A-TPO, TC, HDL-c, LDL-c, SBP, DBP. Slope 1 and slope 2 are the slope coefficients for the segment before and after the inflection point.

### 3.3. Subgroup analyses

**Figure 3:**
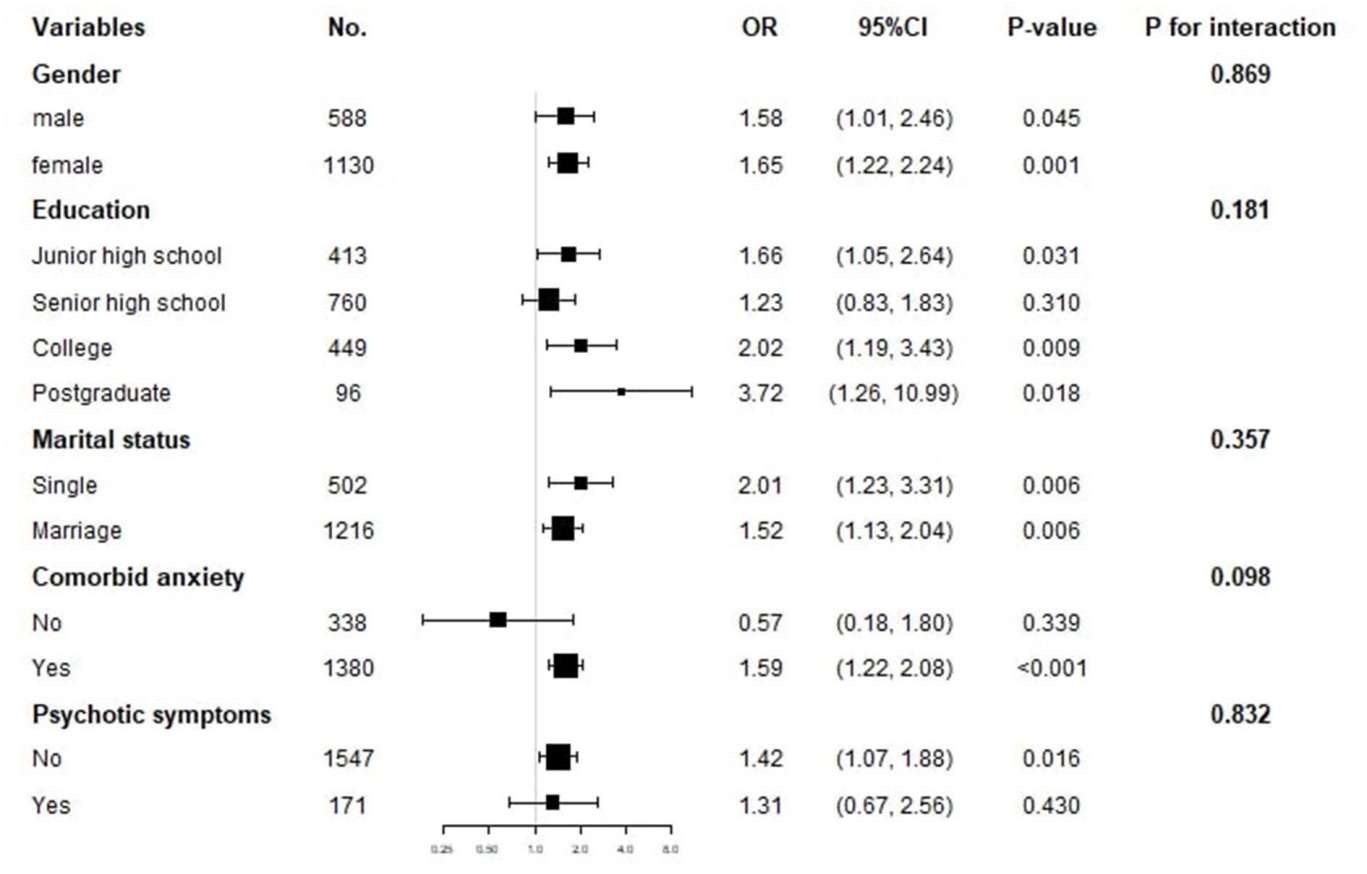
Subgroup analysis of the association between TyG index and suicide attempts. The OR (95% CI) was derived from the Logistic regression model. (Age, sex, education, duration of illness, HAMD, HAMA, TSH, A-TG, A-TPO, TC, HDL-c, LDL-c, SBP and DBP were adjusted).

Figure 3‘s representation of the findings of the subgroup analysis shows a pattern that is constant across subgroups for sex (male, female), marital status (single, married), education (junior high school, senior high school, college, postgraduate), comorbid anxiety (no, yes), and psychotic symptoms (no, yes), with no significant interaction effects observed (all *p* for interaction >0.05).

## 4. Discussion

To the best of our knowledge, this is the first study to examine the relationship between TyG index and SA in a moderately large sample size of patients with FEDN MDD in China. Even after correcting for any confounding factors, our results show a strong correlation between TyG index and SA. Various stratification and sensitivity analysis revealed the connection to be constant. Additionally, TyG index and SA also showed a non-linear connection, with an inflection point of 9.29. No significant relationship was observed on the left side of the inflection point, whereas on the right side of the inflection point, there was a notable positive association between each incremental unit increase in TyG index and a substantial 247% rise in the risk of SA. These results provide novel insights into the link between TyG index and SA in patients with MDD. However, further investigation is required to comprehend the processes underlying this link, and a number of possibilities may be present.

First off, IR has been linked to suicidal behavior and is often associated with blood glucose issues in depressed people. For instance, cross-sectional research in South Korea with over 160,000 participants found that IR was linked to a greater risk of depression, with an increase in incidence of 4% and 17% for young adults and non-diabetics, respectively (35). Furthermore, it has been shown that changes in IR and glucose metabolism are linked to suicidal attempts and ideation in depression (36). On the other side, suicidal thoughts can also cause metabolic problems and impaired lipid synthesis, which can aggravate IR and raise blood sugar levels in type 2 diabetes (37). Excessive fluctuations and uncontrolled blood sugar levels may impair patient awareness of self-management, which is linked to suicidal ideation (38). Our study demonstrated a positive relationship between SA and TyG index, which remained stable even after controlling for relevant confounders and across multiple subgroup analyses. The mechanisms underlying the association between TyG index and SA in patients with MDD require further investigation.

Secondly, the association between a high TyG score and a greater risk of SA may be explained by reverse causation. In addition to IR, a greater TyG index is associated with a variety of harmful health conditions, including metabolic syndrome, obesity, diabetes, hypertension, cerebrovascular disease, and disturbances of the lipid metabolism (39–41). The illness condition may also be correlated with an increased level of depression and SA. The incidence of suicide fatalities was positively connected with those from ischemic heart disease and stroke, according to a 27 year study’s time series between 1979 and 2005 (42). In addition, Hawkins et al. (43) found that those with cardiovascular illness who also had depression had a higher likelihood of having SA in the previous year. Additionally, there is proof that SA, lipid metabolism issues, and metabolic syndrome are related in people with MDD (44, 45). The concept about the glucose-fatty acid cycle is a potential explanation (46, 47). Depressed people are reported to have lower HDL cholesterol and higher triglyceride levels when compared to healthy controls. Triglycerides are likely causal risk factors for depression (48).

Thirdly, the link between TyG index and SA in depression may be influenced by inflammatory and oxidative stress. According to the monoamine hypothesis, lower serotonin levels are connected to a variety of metabolic and mood-related problems (49, 50) and that depression is related to monoaminergic dysfunction. On the other hand, the pro-inflammatory cytokines and immunological activity that cause tryptophan depletion and serotonergic hypofunction are highlighted by the inflammatory theory of depression (51, 52). It is understood that type 2 diabetes is an immune-mediated condition in which cytokines are crucial for insulin signaling and the eradication of beta cells that produce insulin (53). A potential pathophysiological mechanism underpinning IR and altered glucose metabolism in depression and suicidal behavior may be the interaction between serotonin and pro-inflammatory cytokines (54, 55). According to recent studies, suicidal behavior is linked to inflammation in both the peripheral and central neural systems (56). Especially in those with a history of childhood trauma and high inflammation, targeting inflammatory processes may be a unique therapy option for lowering suicide risk (57). TyG index and inflammatory indicators such white blood cells and C-reactive protein have been linked positively in prior research (58). Inflammation, oxidative stress, and endothelial dysfunction are also associated with a high TyG index. Both oxidative stress and inflammation can harm the vascular endothelium, which aids in the emergence of a number of illnesses, such as dementia and depression (59). Patients with MDD who attempted suicide showed higher levels of IL-6, which may be impacted by traumatic and stressful situations, according to recent research (60). The link between TyG index and SA in MDD in this study was only found to be statistically significant when TyG index was larger than 9.29, indicating a threshold impact that calls for more investigation into the underlying mechanism.

The current study has a number of advantages. First off, it concentrates on a research group of people with FEDN MDD, which reduces the impact of confounding variables including disease duration, lifestyle interventions, drugs, and associated medical conditions. Second, the linear and nonlinear correlations between TyG index and SA were investigated using both multivariable logistic regression analysis and smoothing plots. However, the study also has several limitations. First of all, its cross-sectional design prevents it from proving causality. Future research should use longitudinal designs to more fully explore the causal association between TyG index and SA in patients with MDD. Second, only Han Chinese patients were included in the study’s patient population, which was drawn from the mental outpatient department of a general hospital in Taiyuan, Shanxi Province, China. Confirmation of these results in people with various ethnic and clinical backgrounds is required. Thirdly, SA was identified using interviews and medical records, which are not structured assessment tools for suicidal behavior. In addition, we did not have access to detailed information on the severity, plans, or thoughts behind the SA. Therefore, future studies should employ specific suicide questionnaires to collect data on these aspects of SA. Fourthly, the study’s participants were patients with MDD who were having their first episode and who had not sought therapy for their depressive symptoms. It is not possible to exclude patients whose diagnosis may have changed to bipolar disorder, as the initial depressive episode may have mirrored the MDD episode. Fifth, since the patients with MDD included in this study had depression that was fairly severe—the 17-item HAMD scores are higher than or equal to 24, the mean HAMA is over 20—the findings of this study may not be generalizable to patients who are less seriously afflicted. Finally, a number of confounding variables that may have a major influence on the connection between TyG index and SA were not examined, including smoking, drinking, personality characteristics, family income, social position, and biological variables. To further understand the pathophysiological processes behind the link between TyG index and SA in individuals with MDD, future research should take into account more confounding variables.

In conclusion, TyG index and SA had a nonlinear association in Chinese patients with FEDN MDD, with an inflection point around 9.29. On the right side of the inflection point, but not on the left, there was a positive correlation between TyG index and SA. The findings should be regarded as preliminary and verified by follow-up research employing a longitudinal design and structured evaluation instruments due to the poor knowledge of the underlying processes, the cross-sectional design, and other limitations of this study.

## Data availability statement

The raw data supporting the conclusions of this article will be made available by the authors, without undue reservation.

## Ethics statement

The studies involving human participants were reviewed and approved by the Institutional Review Board (IRB) of Shanxi Medical University (No. 2016-Y27) and performed in accordance with the Declaration of Helsinki. The patients/participants provided their written informed consent to participate in this study.

## Author contributions

XyZ, XD, and JL: study design. JL, XmZ, HY, and YL: investigation. JL, XbZ, QW, and ZL: analysis and interpretation of data. JL, XmZ, YL, and FJ: drafting of the manuscript. XD and XyZ: critical revision of the manuscript. JL, XmZ, YL, XD, and XyZ: approval of the final version for publication. All authors contributed to the article and approved the submitted version.

## Funding

This work was supported by the Gusu Talent Program (nos. GSWS2021052, GSWS2021053, and GSWS2019070), Key Diagnosis and Treatment Program of Suzhou (LCZX202016), the Suzhou Clinical Medical Center for Mood Disorders (Szlcyxzx202109), the Medical Science and Technology Development Foundation, Nanjing Department of Health (nos. YKK21216, YKK20184, and YKK22264). The funding sources of this study had no role in study design, data collection and analysis, decision to publish, or preparation of the article.

## Conflict of interest

The authors declare that the research was conducted in the absence of any commercial or financial relationships that could be construed as a potential conflict of interest.

## Publisher’s note

All claims expressed in this article are solely those of the authors and do not necessarily represent those of their affiliated organizations, or those of the publisher, the editors and the reviewers. Any product that may be evaluated in this article, or claim that may be made by its manufacturer, is not guaranteed or endorsed by the publisher.
